# The Influence of Temperature on the Larval Development of *Aelurostrongylus abstrusus* in the Land Snail *Cornu aspersum*

**DOI:** 10.3390/pathogens10080960

**Published:** 2021-07-29

**Authors:** Simone Morelli, Mariasole Colombo, Anastasia Diakou, Donato Traversa, Marika Grillini, Antonio Frangipane di Regalbono, Angela Di Cesare

**Affiliations:** 1 Faculty of Veterinary Medicine, University Teaching Veterinary Hospital, University of Teramo, 64100 Teramo, Italy; smorelli@unite.it (S.M.); mcolombo@unite.it (M.C.); dtraversa@unite.it (D.T.); 2 School of Veterinary Medicine, Aristotle University of Thessaloniki, 54124 Thessaloniki, Greece; diakou@vet.auth.gr; 3 Department of Animal Medicine, Production and Health, University of Padova, 35020 Legnaro, Italy; marika.grillini@phd.unipd.it (M.G.); antonio.frangipane@unipd.it (A.F.d.R.)

**Keywords:** *Aelurostrongylus abstrusus*, *Cornu aspersum*, development, temperature

## Abstract

The metastrongyloid *Aelurostrongylus* *abstrusus* has an indirect lifecycle involving gastropod intermediate hosts. The widespread snail *Cornu* *aspersum* is an efficient intermediate host of *A. abstrusus*. As the temperature may influence the developmental rate of metastrongyloids from first (L1) to the third infective larval stage (L3) inside molluscs, this study evaluated the effect of two controlled temperatures on the development of *A. abstrusus* in *C. aspersum*. Overall, 300 snails were infected with 500 L1 of *A. abstrusus* and kept at ∼25 °C. Fifteen days post infection (D15), the overall developmental rate to L3 (0.8%) was assessed in a subset of 20 snails. The remaining gastropods were divided in 2 groups, i.e., 180 still kept at ∼25 °C (G1) and 100 hibernated at ∼4 °C (G2). On D30, the larval development was evaluated in 20 snails from each group, while another batch of 80 snails was selected random from G1 and hibernated at ∼4 °C (G3). The larval developmental rate was determined digesting 20 snails from each of the three groups on D45, D60, and D75. The higher mean developmental rate was registered in G1 (3.8%) compared to G2 (1.9%) and G3 (2.3%), indicating that the development to L3 of *A. abstrusus* in *C. aspersum* is positively influenced by the increase of temperature.

## 1. Introduction

Adult stages of the cat lungworm *Aelurostrongylus abstrusus* (Metastrongyloidea, Angiostrongylidae) inhabit bronchioles, alveolar ducts, and alveoli of domestic cats (i.e., the natural host) and some wild felids [1,2,3]. Felid aelurostrongylosis is distributed worldwide, with most records from Europe [4,5,6] and less common descriptions in the Americas [7,8] and Africa [9].

Clinical signs in infected cats vary from subtle to severe and include ocular and nasal discharge, sneezing, wheezing, dyspnea, cough, and open-mouth breath [10].

The life cycle of *A. abstrusus* is indirect and requires snails and slugs as intermediate hosts. The first stage larvae (L1) are shed in the environment through the respiratory mucous or the faeces of the infected cat, penetrate or are ingested by gastropods, and reach the infective stage (third stage larvae, L3), after a time period that varies depending on environmental factors and is estimated to be about 11–18 days under optimum conditions [11,12,13]. Cats acquire the infection by ingesting intermediate, or more commonly, paratenic hosts, e.g., birds, small rodents, and reptiles [3].

The epizootiology of mollusk-borne parasitosis, including aelurostrongylosis, is influenced by extrinsic and intrinsic factors. Some, e.g., climate changes, have an impact on vector phenology and ecology, enhancing the presence of mollusks and infective stages of parasites in endemic areas, while others have the potential to spur the geographic distribution of transmitted pathogens, as in the case of globalization and global transports, anthropization of wildlife habitats, and movements of animals from enzootic to non-enzootic regions [14]. Accordingly, geographic distribution, spreading, and developmental and reproduction rates of snails and slugs may have a key role in increasing numbers of documented cases of cat aelurostrongylosis [14,15].

Experimental studies have shown that many genera of molluscs act as intermediate hosts of *A. abstrusus*, [11,12,16,17,18] and wild-caught gastropods infected by larvae of *A. abstrusus* have been found in several countries [19,20,21,22,23,24].

The land snail *Cornu aspersum* is a globally invasive species widespread in Europe, whose distribution is favored by heliciculture for human consumption [16,25,26]. Studies in natural and experimental settings have proven its ability to act as intermediate host of major metastrongyloid nematodes, including *A. abstrusus* [11,13,16,27]. Accordingly, cats infected by L3 harvested from experimentally infected *C. aspersum* develop patent aerulostrongylosis [16,28,29]. 

Laboratory studies on the larval development of *A. abstrusus* in *C. aspersum* have been conducted under (completely or partially) uncontrolled conditions (e.g., temperature, humidity) [11,13,16]; thus, information on environmental factors impacting on biological features and larval development are poorly known. Data from the last decade have suggested that environmental temperature influences survival, vitality, and/or developmental rate of metastrongyloid lungworms in the environment and/or in the molluscs [30,31,32].

The present study has evaluated the influence of environmental temperature on larval development of *A. abstrusus* in *C. aspersum* in comparison with data recently gathered on the larval development of the closely related felid lungworm *Troglostrongylus brevior* (Metastrongyloidea, Crenosomatidae) under the same experimental conditions [31]. Biological and ecological implications on the influence of temperature on the larval development of *A. abstrusus* are discussed.

## 2. Results

**Figure 1:**
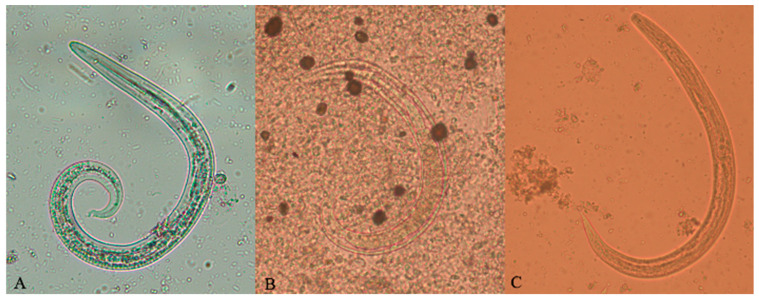
**Larvae of *Aelurostrongylus abstrusus*.** First (**A**), second (**B**), and third (**C**) stage larvae of *Aelurostrongylus abstrusus*, detected in the sediment obtained from the Baermann technique (**A**) and from snail digestion (**B**,**C**).

No pre-existing infections were recorded in the snails microscopically and genetically examined prior to the study. The digestion performed at D15 showed an overall larval development approximately of 1.9% and 0.8% for L2 and L3, respectively. L1 were detected with a percentage of 2.8% on D15 and of 0.6% and 0.2% on D30 in G1 (i.e., snails maintained at environmental conditions in *vivaria,* i.e., 25 ± 2 °C temperature, ∼80% humidity) and G2 (i.e., snails hibernated at 4 ± 2 °C on D15), respectively, while no L1 were present from D45 to D75 (Figure 1).

L2 and L3 were recorded at all time points of all groups, i.e., D30, D45, D60, and D75 in G1 and G2 and D45, D60, and D75 in G3 (i.e., snails hibernated at 4 ± 2 °C on D30) (Figure 1). Rates of larval development detected at D30, D45, D60, and D75 were 2.8%, 1.4%, 6.0%, and 5.0% (mean 3.8%) for G1 and 3.2%, 2.8%, 1.0%, and 0.6% (mean 1.9%) for G2. The percentage of L3 retrieved in snails of G3 were 2.0%, 3.5%, and 1.5% (mean 2.3%) on days 45, 60, and 75 p.i., respectively.

Detailed information on each larval stage found in G1, G2, and G3 at each time-point is listed in Table 1. All molecular examinations confirmed the identity of the retrieved larvae as *A. abstrusus.*

## 3. Discussion

The overall number of larvae retrieved in snails herein examined could have been influenced by the fact that only the muscular foot (and not the whole body) was digested. Although most metastrongyloid larvae localize in the mollusc foot, a varying number of larvae can be present in the viscera [16,33,34]. In the present study, only the muscular foot was digested to compare data with those obtained in a recent experiment which investigated the larval development rate of *T. brevior* in snails kept at environmental temperature (25 ± 2 °C) vs. snails hibernated at 4 ± 2 °C [31]. In the present study, G2 was set up for a direct comparison with data from this latter study, while snails of G3 were later hibernated for a further biological comparison between these two lungworms, as the development of *A. abstrusus* to L3 inside molluscs is slower than that of *T. brevior* and requires averagely twice as much time [16,31]. 

The timepoints of digestion were selected based on previous studies evaluating the larval development of *Angiostrongylus* spp., *T. brevior* and *A. abstrusus*, in *C. aspersum* [16,31,35]. In particular, 75 days were selected as the length of the study period and the timepoints for the snail examinations were appointed with an interval of 15 days to make the data comparable with those of other studies [13,16,31,35,36].

The present data are in accordance with past information, i.e., the first *A. abstrusus* L3 appear 11–18 days p.i. in intermediate hosts experimentally infected and kept at environmental conditions of 18–30 °C [11,12,13]. On the other hand, in terms of early detection of L3, the results of the present study are in contrast with those of another experimental study [16], where the first L3 were detected 21 days p.i. in experimentally infected snails kept at variable environmental temperature from 18.8 to 29.5 °C. Moreover, in this latter experiment, a developmental rate of 47.9%, i.e., higher of the here-presented data, was registered. It is hard to provide a sound comparison of the present data with those previously presented [16] because in that older study, snails were infected with a lower dose of 100 L1 and then kept at uncontrolled temperatures reaching 29.5 °C, i.e., values possibly promoting the molt of L2 to L3. In fact, 30 °C is considered the optimum temperature for larval development, while at lower temperatures, the larval development is reduced [11]. 

**Figure 2:**
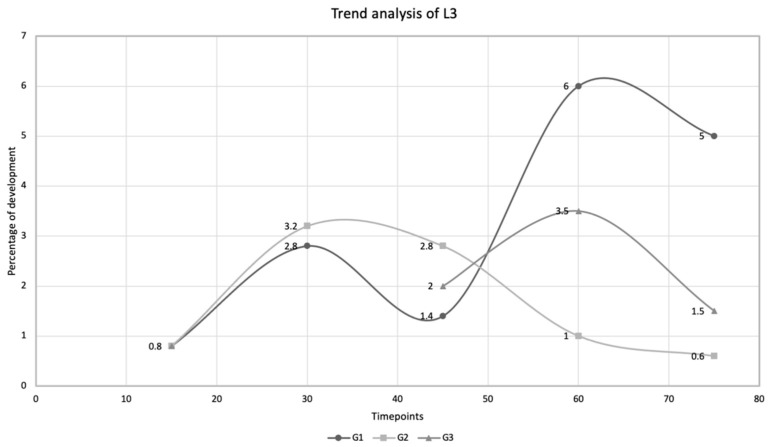
Trend analysis of *Aelurostrongylus abstrusus* larval development rate to the third larval stage (L3) in *Cornu aspersum* snails experimentally infected with 500 first-stage larvae on day 0. Percentage of development (%) to L3 at different timepoints, i.e., day 15, day 30, day 45, day 60, and day 75 post infection, detected in snails kept at 25 ± 2 °C (G1), in snails hibernated at 4 ± 2 °C on D15 post infection (G2), and in snails hibernated at 4 ± 2 °C on D30 post infection (G3).

G1 has shown an overall increasing trend, though not linear, in the number of L3 (Figure 2). The percentage of L3 herein found on D45 is unexpectedly lower than those detected on D30 and D60. This could be due to unpredictable shortcomings of the artificial digestion performed on D45, as the same unexpected results have also been observed in the number of L2 detected in G1 on D45. As the aim of this study was to evaluate the general biological effect of temperature on the larval development of *A. abstrusus*, this has been considered to have had no impact on the reliability of the present results.

**Figure 3:**
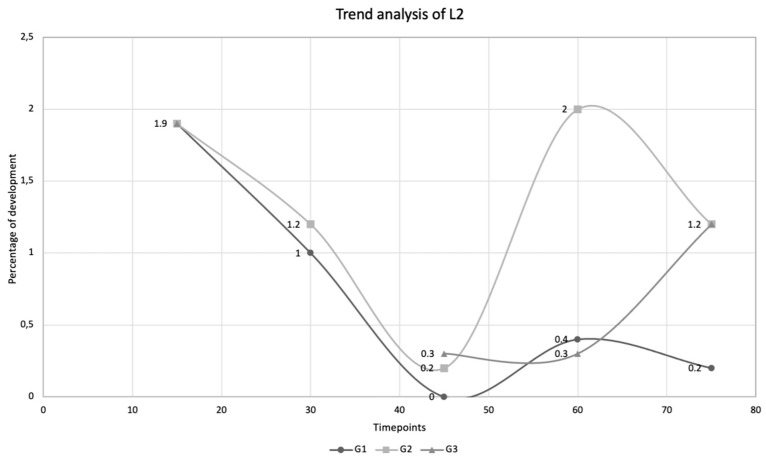
Trend analysis of *Aelurostrongylus abstrusus* larval development to the second larval stage (L2) in *Cornu aspersum* snails experimentally infected with 500 first-stage larvae on day 0. Percentage of development (%) to L2 at different timepoints, i.e., day 15, day 30, day 45, day 60, and day 75 post infection, detected in snails kept at 25 ± 2 °C (G1), in snails hibernated at 4 ± 2 °C on D15 post infection (G2), and in snails hibernated at 4 ± 2 °C on D30 post infection (G3).

Overall, this study suggests that *A. abstrusus* L2 easily molt to L3 when exposed to favorable temperatures of 25 ± 2 °C, whilst they probably stop or slow down their development when exposed to lower temperatures. This is indicated by (i) the decreasing linear trend in the number of L3 in G2, (ii) the higher number of L2 in snails of G2 compared to G1, and (iii) the decreasing number of L2 in G1 (Figure 3). Even if not directly comparable, data on the development from L2 to L3 are partially in line with those reported by Di Cesare et al. (2013) [16], where *A. abstrusus* L2 were found until 65 days p.i. in snails kept at 6.7–22 °C, while they were absent at 58 days p.i. in snails kept at 18.8 to 29.5 °C. The absence of L2 at 58 days p.i. could be explained by the higher temperatures reached during the experiment favoring the molt from L2 to L3 [16], as indicated by the high percentage of L3 retrieved in the same study. 

Some results here were unexpected: (i) the initial increase of L3 developmental rate post hibernation from D45 to D60, followed by a reduction on D75 in G3 and (ii) the higher L3 developmental rate in G2 vs. G1 on D30 and the subsequent reduction from D45 to D75 (Figure 2). This could be explained by the effect of snail hibernation on larval development of metastrongyloids. It has been recently shown that *T. brevior* is able to increase its larval development rate to L3 in *C. aspersum* hibernated at 4 ± 2 °C [31]. It is here suggested that hibernation may transiently favor the development of *A. abstrusus* to the infective stage in *C. aspersum*. Nonetheless, while for *T. brevior* hibernation leads to a progressive rise in the developmental rate of larvae [31], lower temperatures promote larval development only in the early phases for *A. abstrusus*. This variability could be explained by factors related to host-parasite interactions, as previously discussed for *T. brevior* [31]. In the case of adverse environmental conditions (like low temperatures), snails start hibernation, which is a dormancy state characterized by reduction of oxygen consumption and water loss [37,38]. This overwintering strategy reduces mortality and improves reproductive chances [37,39,40]. During hibernation, the snail glucose requirements are reduced due to the hypometabolism [41]. It has been recently hypothesized that under these circumstances, *T. brevior* larvae benefit from more available nutrients and reach the infective stage in high numbers [31]. This does not occur for larvae of *A. abstrusus*, which develop and molt to infective stage only for limited periods at low temperatures (Figure 2). It cannot be excluded that a prolonged exposure to colder temperatures progressively slows down the metabolism of *A. abstrusus* larvae inside molluscs and that the same does not occur for *T. brevior*. If so, only a limited number of larvae would be able to reach the infective stage over time despite the high availability of nutrients in hibernated snails. 

A recent study demonstrated that L1 of *A. abstrusus* maintained in water at 4 °C ± 1 °C can survive for longer period (i.e., 231 days) if compared to L1 kept at higher temperatures, i.e., 70 days at 14 °C and 42 days at 28 °C [32]. Moreover, refrigerated L1 of *A. abstrusus* resulted more efficient in infecting and developing inside *C. aspersum* if compared to L1 kept at −20 ± 1 °C, 14 ± 1 °C and 28 ± 1 °C prior to the snail infection [32]. A direct comparison between results from [32] and those of the present study are difficult as in the former experiment because (i) the snails were repeatedly infected with 300 L1 kept at different temperatures by direct injection in the muscular foot every 21 days and (ii) once infected, snails were kept only at environmental temperature (i.e., 20 ± 3 °C). However, it seems that lower temperatures have a positive effect on the first two larval stages of *A. abstrusus* rather than on the molt to the infective stage. This biological feature could be a strategy developed by *A. abstrusus* to ensure the infection of the intermediate host. In fact, this lungworm is enzootic with high prevalence in temperate areas, e.g., the Mediterranean basin, where during winter, these temperatures are often reached, and in northern Europe, where the climate is colder as well [4,5,42,43,44,45]. Data on the development to L3 of *T. brevior* [31] vs. *A. abstrusus* ([16], present study) and the less frequent detection of the latter in colder climates and at higher altitudes (e.g., northern countries) indicate that crenosomatids have greater cold-resistance abilities than angiostrongylids [16,31,46,47,48,49]. Studies have also shown higher survival abilities at freezing temperatures of the L1 of the canine crenosomatid *Crenosoma vulpis* vs. the canine angiostrongylid *Angiostrongylus vasorum* [46,47]. It is however important to note that data gathered from studies on the environmental survival of *A. abstrusus* and *T. brevior* L1 at different temperatures are contrasting. The results of two studies have initially suggested a higher resistance of *A. abstrusus* vs. *T. brevior* at both refrigeration and room temperature, as L1 of *T. brevior* survived at 4 °C ± 1 for shorter periods than *A. abstrusus* L1, i.e., 142 vs. 231 days, while at 26 °C, *T. brevior* L1 died in seven days and those of *A. abstrusus* survived for 42 days [32,50]. There is also the evidence that *T. brevior* is more resistant to cold than *A. abstrusus*. While in the experiment from [32], the L1 of *A. abstrusus* survived 28 days at −20 ± 1 °C, other data suggest that L1 of *T. brevior* can survive longer (i.e., 165 days) than those of *A. abstrusus* (i.e., 40 days) [51]. Further standardized experiments are thus needed to ultimately elucidate differences in terms of cold resistance of L1 of *T. brevior* and *A. abstrusus* and to understand potential epizootiological implications. This knowledge is useful for elucidating drivers which influence epizootiological patterns in different geographies and possible seasonality of aelurostrongylosis and troglostrongylosis. 

Under an epizootiological standpoint, it should be taken into account that a single specimen of *C. aspersum* might represent a source of lungworm infection for both dogs and cats, as it is proven that this snail can harbor and sustain the development of more than one nematode at the same time [13,52]. Accordingly, mixed infections by *A. abstrusus* and *T. brevior* are often reported both in naturally infected intermediate and definitive hosts [6,13,21,53]. Further studies aiming at clarifying whether temperature can favor the development in the intermediate host of one species rather than another are warranted to better understand the possible epizootiological implications in terms of seasonality and geographical distribution of metastrongyloid lungworms.

In conclusion, despite the fact that development to L3 of *A. abstrusus* is positively influenced by the increase of temperature, this parasite is able to develop also at refrigeration temperatures though with lower efficiency than *T. brevior*, confirming high adaptation capacities to different environmental conditions [32]. Temperature and global warming in particular could play a central role in the spreading of the *A. abstrusus* because other than enhancing its development inside intermediate hosts, it is also implicated in the progressive diffusion of gastropods [54]. Knowledge on the interactions between lungworms and their intermediate hosts is still to be generated, and future experimental and field studies on this topic are herein encouraged. Such studies may provide useful data for the establishment of new monitoring and prevention plans for cat aelurostrongylosis but also for other gastropod-borne diseases of veterinary interest, e.g., feline troglostrongylosis and canine angiostrongylosis.

## 4. Materials and Methods

Thirty hundred and twenty specimens of adult *C. aspersum* were purchased from a snail farm intended for human consumption in Italy. Before starting the trial, 20 snails, selected as a representative batch, were confirmed to be negative for pre-existing nematode infections by microscopy after artificial digestion and sediment examination under a light microscope (Axioscope 40, Zeiss, Oberkochen, Germany) [31] and by molecular examinations using a triplex PCR protocol to detect rDNA ITS2 of *A. abstrusus*, *T. brevior,* and *Angiostrongylus chabaudi* [55,56].

A clinically healthy cat naturally infected by *A. abstrusus* was selected as donor of *A. abstrusus* L1 with the consent of the owner. Nine consecutive defecations over four days were used to obtain the L1 with the Baermann’s method [57]. After the collection was completed, the cat was treated with an anthelmintic labelled for *A. abstrusus* by a referring veterinarian. L1 were microscopically and genetically confirmed to be *A. abstrusus* [14,56].

On study day 0 (D0), 100 μL of Baermann sediment containing 500 L1 of *A. abstrusus* were administered to each of 300 snails using infection chambers containing six wells that were filled with potato slices. The infective doses were placed on the surface of the potato, and the snails were put at direct contact with the larvae as previously described [16]. Snails were kept in *vivaria* at 25 ± 2 °C temperature, ∼80% humidity, and fed twice a week. Fifteen days post infection (D15), 20 snails were randomly selected and examined via artificial digestion [31] to assess the overall larval developmental rate. The whole sediment obtained was observed under a light optical microscope and the different larval stages of *A. abstrusus* detected (L1, L2, and L3) were counted and identified using morphological and morphometrical keys as previously described [13]. Once obtained, the number of viable L1, L2, and L3, the following formula has been used to calculate the developmental rate:

[n of larvae (L2 or L3) retrieved/infective dose (500 L1)] × 100 = developmental rate (%)].

Based on a random selection, on the same day, the remaining 280 snails were divided in two groups: 180 snails were maintained at environmental conditions in *vivaria*, i.e., at 25 ± 2 °C with ∼80% humidity (G1), and 100 snails were hibernated at 4 ± 2 °C (G2).

On day 30 post infection (D30), 120 snails were randomly selected, i.e., 20 snails from non-hibernated (G1) group and 20 snails from hibernated (G2) group, and were examined by artificial digestion, and 80 snails from G1 were hibernated at 4 ± 2 °C, setting up the group 3 (i.e., G3—snails hibernated at 4 ± 2 °C on D30 post infection).

On day 60 and day 75 post infection (D60 and D75), twenty snails from each of the three groups were randomly selected and examined by artificial digestion to evaluate the larval development of *A. abstrusus*. In particular, the snails were examined at each timepoint as a pooled sample/group in a single artificial digestion procedure, counting all larvae at different stages detected and dividing the total number by 20 to obtain (i) a mean value of larvae (L1, L2, and L3) per single snail and (ii) a mean value of developmental rate using the above-mentioned formula.

An aliquot of the sediment obtained from each digestion timepoint was subjected to molecular analysis to confirm the identity of the larvae [55,56].

Of the remaining 60 snails, 23 were considered unsuitable for the experiment (e.g., broken and too big or too small for the infection procedure), and 37 died during the study period.

## Figures and Tables

**Table 1:** Developmental rate (%) at different timepoints of different larval stages (from L1 to L3) of *Aelurostrongylus abstrusus* in *Cornu aspersum* snails infected with a dose of 500 L1, i.e., day 15 (D15), day 30 (D30), day 45 (D45), day 60 (D60), and day 75 (D75) post infection. Snails were randomly divided in three group, i.e., G1—kept at 25 ± 2 °C with ∼80% humidity, G2—hibernated at 4 ± 2 °C on D15 post infection, and G3—hibernated at 4 ± 2 °C on D30 post infection.

Time Points	Larval Stages								
	L1 (%)			L2 (%)			L3 (%)		
**D15**	2.8			1.9			0.8		
	**G1**	**G2**	**G3**	**G1**	**G2**	**G3**	**G1**	**G2**	**G3**
**D30**	0.6	0.2	0	1.0	1.2	0	2.8	3.2	0
**D45**	0	0	0	0	0.2	0.3	1.4	2.8	2.0
**D60**	0	0	0	0.4	2.0	0.3	6.0	1.0	3.5
**D75**	0	0	0	0.2	1.2	1.2	5.0	0.6	1.5

## Data Availability

All the data generated during this work are reported in the manuscript.

## References

[1] Stevanović O., Diakou A., Morelli S., Paraš S., Trbojević I., Nedić D., Sladojević Ž., Kasagić D., Di Cesare A. (2019). Severe Verminous Pneumonia Caused by Natural Mixed Infection with *Aelurostrongylus abstrusus* and *Angiostrongylus chabaudi* in a European Wildcat from Western Balkan Area. Acta Parasitol..

[2] Diakou A., Dimzas D., Astaras C., Savvas I., Di Cesare A., Morelli S., Neofitos Κ., Migli D., Traversa D. (2020). Clinical investigations and treatment outcome in a European wildcat (*Felis silvestris silvestris*) infected by cardio-pulmonary nematodes. Vet. Parasitol. Reg. Stud. Rep..

[3] Traversa D., Morelli S., Di Cesare A., Diakou A. (2021). Felid Cardiopulmonary Nematodes: Dilemmas Solved and New Questions Posed. Pathogens.

[4] Giannelli A., Capelli G., Joachim A., Hinney B., Losson B., Kirkova Z., René-Martellet M., Papadopoulos E., Farkas R., Napoli E. (2017). Lungworms and gastrointestinal parasites of domestic cats: A European perspective. Int. J. Parasitol..

[5] Traversa D., Morelli S., Cassini R., Crisi P.E., Russi I., Grillotti E., Manzocchi S., Simonato G., Beraldo P., Viglietti A. (2019). Occurrence of canine and feline extra-intestinal nematodes in key endemic regions of Italy. Acta Trop..

[6] Morelli S., Diakou A., Di Cesare A., Schnyder M., Colombo M., Strube C., Latino R., Traversa D. (2020). Feline lungworms in Greece: Copromicroscopic, molecular and serological study. Parasitol. Res..

[7] Carruth A.J., Buch J.S., Braff J.C., Chandrashekar R., Bowman D.D. (2019). Distribution of the feline lungworm *Aelurostrongylus abstrusus* in the USA based on fecal testing. J. Feline Med. Surg. Open Rep..

[8] da Silva Lima W., Ferreira Farago E.C., do Nascimento Mesquita M., Duarte Pacheco A., Fernandes Nunes da Silva Malavazi P., Salvador Oliveira H., Morelli S., Colombo M., Di Cesare A., Figueiredo de Souza S. (2021). First Case of Clinical Cat Aelurostrongylosis in the Brazilian Amazon: Clinical and Molecular Insights. Pathogens.

[9] Di Cesare A., Laiacona F., Iorio R., Marangi M., Menegotto A. (2016). *Aelurostrongylus abstrusus* in wild felids of South Africa. Parasitol. Res..

[10] Morelli S., Diakou A., Colombo M., Di Cesare A., Barlaam A., Dimzas D., Traversa D. (2021). Cat Respiratory Nematodes: Current Knowledge, Novel Data and Warranted Studies on Clinical Features, Treatment and Control. Pathogens.

[11] Gerichter C.B. (1949). Studies on the nematodes parasitic in the lungs of Felidae in Palestine. Parasitology.

[12] López C., Panadero R., Paz A., Sánchez-Andrade R., Díaz P., Díez-Baños P., Morrondo P. (2005). Larval development of *Aelurostrongylus abstrusus* (Nematoda, Angiostrongylidae) in experimentally infected *Cernuella* (*Cernuella*) *virgata* (Mollusca, Helicidae). Parasitol Res..

[13] Giannelli A., Ramos R.A., Annoscia G., Di Cesare A., Colella V., Brianti E., Dantas-Torres F., Mutafchiev Y., Otranto D. (2014). Development of the feline lungworms *Aelurostrongylus abstrusus* and *Troglostrongylus brevior* in *Helix aspersa* snails. Parasitology.

[14] Traversa D., Di Cesare A. (2016). Diagnosis and management of lungworm infections in cats: Cornerstones, dilemmas and new avenues. J. Feline Med. Surg..

[15] Di Cesare A., Veronesi F., Traversa D. (2015). Felid Lungworms and Heartworms in Italy: More Questions than Answers?. Trends Parasitol..

[16] Di Cesare A., Crisi P.E., Di Giulio E., Veronesi F., Frangipane di Regalbono A., Talone T., Traversa D. (2013). Larval development of the feline lungworm *Aelurostrongylus abstrusus* in *Helix aspersa*. Parasitol. Res..

[17] Cardillo N.M., Ercole M., Fariña F., Pasqualetti M., Loiza Y., Pérez M., Bonboni A., Ribicich M. (2018). Larval development of *Aelurostrongylus abstrusus* in experimentally infected *Rumina decollata* snails. Vet. Parasitol..

[18] Zottler E., Schnyder M. (2016). Larval development of the cat lungworm *Aelurostrongylus abstrusus* in the tropical freshwater snail *Biomphalaria glabrata*. Parasitol. Open.

[19] Jeżewski W., Buńkowska-Gawlik K., Hildebrand J., Perec-Matysiak A., Laskowski Z. (2013). Intermediate and paratenic hosts in the life cycle of *Aelurostrongylus abstrusus* in natural environment. Vet. Parasitol..

[20] Lange M.K., Penagos-Tabares F., Hirzmann J., Failing K., Schaper R., Van Bourgonie Y.R., Backeljau T., Hermosilla C., Taubert A. (2018). Prevalence of *Angiostrongylus vasorum*, *Aelurostrongylus abstrusus* and *Crenosoma vulpis* larvae in native slug populations in Germany. Vet. Parasitol..

[21] Penagos-Tabares F., Lange M.K., Vélez J., Hirzmann J., Gutiérrez-Arboleda J., Taubert A., Hermosilla C., Chaparro Gutiérrez J.J. (2019). The invasive giant African snail *Lissachatina fulica* as natural intermediate host of *Aelurostrongylus abstrusus*, *Angiostrongylus vasorum*, *Troglostrongylus brevior*, and *Crenosoma vulpis* in Colombia. PLoS Negl. Trop. Dis..

[22] Penagos-Tabares F., Groß K.M., Hirzmann J., Hoos C., Lange M.K., Taubert A., Hermosilla C. (2020). Occurrence of canine and feline lungworms in *Arion vulgaris* in a park of Vienna: First report of autochthonous *Angiostrongylus vasorum*, *Aelurostrongylus abstrusus* and *Troglostrongylus brevior* in Austria. Parasitol. Res..

[23] Fuehrer H.P., Morelli S., Bleicher J., Brauchart T., Edler M., Eisschiel N., Hering T., Lercher S., Mohab K., Reinelt S. (2020). Detection of *Crenosoma* spp., *Angiostrongylus vasorum* and *Aelurostrongylus abstrusus* in Gastropods in Eastern Austria. Pathogens.

[24] Dimzas D., Morelli S., Traversa D., Di Cesare A., Van Bourgonie Y.R., Breugelmans K., Backeljau T., Frangipane di Regalbono A., Diakou A., Backeljau T. (2020). Intermediate gastropod hosts of major feline cardiopulmonary nematodes in an area of wildcat and domestic cat sympatry in Greece. Parasites Vectors.

[25] Global Invasive Species Database. www.iucngisd.org.

[26] Guiller A., Martin M.C., Hiraux C., Madec L. (2012). Tracing the invasion of the mediterranean land snail *Cornu aspersum* aspersum becoming an agricultural and garden pest in areas recently introduced. PLoS ONE.

[27] Helm J., Roberts L., Jefferies R., Shaw S.E., Morgan E.R. (2015). Epidemiological survey of *Angiostrongylus vasorum* in dogs and slugs around a new endemic focus in Scotland. Vet. Rec..

[28] Schnyder M., Di Cesare A., Basso W., Guscetti F., Riond B., Glaus T., Crispi P., Deplazes P. (2014). Clinical, laboratory and pathological findings in cats experimentally infected with *Aelurostrongylus abstrusus*. Parasitol. Res..

[29] Raue K., Rohdich N., Hauck D., Zschiesche E., Morelli S., Traversa D., Di Cesare A., Roepke R.K.A., Strube C. (2021). Efficacy of Bravecto^®^ Plus spot-on solution for cats (280 mg/ml fluralaner and 14 mg/ml moxidectin) for the prevention of aelurostrongylosis in experimentally infected cats. Parasites Vectors..

[30] Gökpinar S., Yildiz K. (2010). The effect of different temperatures on viability of *Aelurostrongylus abstrusus* first stage larvae in faeces of cats. Turk. Parazitol. Derg..

[31] Morelli S., Traversa D., Colombo M., Raue K., Strube C., Pollmeier M., Di Cesare A. (2020). The effect of the hibernation on the larval development of *Troglostrongylus brevior* in the land snail *Cornu aspersum*. Vet. Parasitol..

[32] Napoli E., Arfuso F., Gaglio G., Abbate J.M., Giannetto S., Brianti E. (2020). Effect of different temperatures on survival and development of *Aelurostrongylus abstrusus* (Railliet, 1898) larvae. J. Helminthol..

[33] Mendonca C.L., Carvalho O.S., Mota E.M., Pelajo-Machado M., Caputo L.F., Lenzi H.L. (1999). Penetration sites and migratory routes of *Angiostrongylus costaricensis* in the experimental intermediate host (*Sarasinula marginata*). Mem. Inst. Oswaldo Cruz.

[34] Giannelli A., Colella V., Abramo F., do Nascimento Ramos R.A., Falsone L., Brianti E., Varcasia A., Dantas-Torres F., Knaus M., Fox M.T. (2015). Release of lungworm larvae from snails in the environment: Potential for alternative transmission pathways. PLoS Negl. Trop. Dis..

[35] Di Cesare A., Crisi P.E., Bartolini R., Iorio R., Talone T., Filippi L., Traversa D. (2015). Larval development of *Angiostrongylus vasorum* in the land snail *Helix aspersa*. Parasitol. Res..

[36] Colella V., Mutafchiev Y., Cavalera M.A., Giannelli A., Lia R.P., Dantas-Torres F., Otranto D. (2016). Development of *Crenosoma vulpis* in the common garden snail *Cornu aspersum*: Implications for epidemiological studies. Parasites Vectors.

[37] Ansart A., Vernon P., Daguzan J. (2001). Photoperiod is the main cue that triggers supercooling ability in the land snail, *Helix aspersa* (Gastropoda: Helicidae). Cryobiology.

[38] Ramos-Vasconcelos G.R., Hermes-Lima M. (2003). Hypometabolism, antioxidant defenses and free radical metabolism in the pulmonate land snail *Helix aspersa*. J. Exp. Biol..

[39] Artacho P., Nespolo R.F. (2009). Natural selection reduces energy metabolism in the garden snail, *Helix aspersa* (*Cornu aspersum*). Evolution.

[40] Nicolai A., Filser J., Briand V., Charrier M. (2010). Seasonally contrasting life-history strategies in the land snail *Cornu aspersum*: Physiological and ecological implications. Can. J. Zool..

[41] Tunholi-Alves V.M., Tunholi V.M., Garcia J., Mota E.M., Castro R.N., Pontes E.G., Pinheiro J. (2018). Unveiling the oxidative metabolism of *Achatina fulica* (Mollusca: *Achatina fulica* (Mollusca: Gastropoda) experimentally infected to *Angiostrongylus cantonensis* (Nematoda: Metastrongylidae). Parasitol. Res..

[42] Rojas M., Li L.Z., Kanakidou M., Hatzianastassiou N., Seze G., Le Treut H. (2013). Winter weather regimes over the Mediterranean region: Their role for the regional climate and projected changes in the twenty-first century. Clim. Dyn..

[43] Diakou A., Di Cesare A., Barros L.A., Morelli S., Halos L., Beugnet F., Traversa S. (2015). Occurrence of *Aelurostrongylus abstrusus* and *Troglostrongylus brevior* in domestic cats in Greece. Parasites Vectors.

[44] Di Cesare A., Di Francesco G., Frangipane di Regalbono A., Eleni C., De Liberato C., Marruchella G., Iorio R., Malatesta D., Romanucci M.R., Bongiovanni L. (2015). Retrospective study on the occurrence of the feline lungworms *Aelurostrongylus abstrusus* and *Troglostrongylus* spp. in endemic areas of Italy. Vet. J..

[45] Di Cesare A., Veronesi F., Grillotti E., Manzocchi S., Perrucci S., Beraldo P., Cazzin S., De Liberato C., Barros L.A., Simonato G. (2015). Respiratory nematodes in cat populations of Italy. Parasitol. Res..

[46] Ferdushy T., Kapel C.M., Webster P., Al-Sabi M.N., Grønvold J.R. (2010). The effect of temperature and host age on the infectivity and development of *Angiostrongylus vasorum* in the slug Arion lusitanicus. Parasitol. Res..

[47] Hodžić A., Alić A., Klebić I., Kadrić M., Brianti E., Duscher G.G. (2016). Red fox (*Vulpes vulpes*) as a potential reservoir host of cardiorespiratory parasites in Bosnia and Herzegovina. Vet. Parasitol..

[48] Grandi G., Comin A., Ibrahim O., Schaper R., Forshell U., Lind E.O. (2017). Prevalence of helminth and coccidian parasites in Swedish outdoor cats and the first report of *Aelurostrongylus abstrusus* in Sweden: A coprological investigation. Acta Vet. Scand..

[49] Gueldner E.K., Gilli U., Strube C., Schnyder M. (2019). Seroprevalence, biogeographic distribution and risk factors for *Aelurostrongylus abstrusus* infections in Swiss cats. Vet. Parasitol..

[50] Ramos R.A., Giannelli A., Dantas-Torres F., Brianti E., Otranto D. (2013). Survival of first-stage larvae of the cat lungworm *Troglostrongylus brevior* (Strongylida: Crenosomatidae) under different conditions. Exp. Parasitol..

[51] Traversa D. (2021). Personal communication.

[52] Dimzas D., Diakou A., Di Cesare A., Van Bourgornie Y.R., Backeliau T., Staikou A., Traversa D. Gastropods as intermediate hosts of feline cardio-pulmonary parasites in Greece: Preliminary results. Proceedings of the 14th International Congress on the Zoogeography and Ecology of Greece and Adjacent Regions.

[53] Di Cesare A., Frangipane di Regabono A., Tessarin C., Seghetti M., Iorio R., Simonato G., Traversa D. (2014). Mixed infection by *Aelurostrongylus abstrusus* and *Troglostrongylus brevior* in kittens from the same litter in Italy. Parasitol. Res..

[54] Proćków M., Konowalik K., Proćków J. (2019). Contrasting effects of climate change on the European and global potential distributions of two Mediterranean helicoid terrestrial gastropods. Reg. Environ. Chang..

[55] Di Cesare A., Morelli S., Colombo M., Simonato G., Veronesi F., Marcer F., Diakou A., D’Angelosante R., Pantchev N., Psaralexi E. (2020). Is Angiostrongylosis a Realistic Threat for Domestic Cats?. Front. Vet. Sci..

[56] Di Cesare A., Veronesi F., Frangipane di Regalbono A., Iorio R., Traversa D. (2015). Novel Molecular Assay for Simultaneous Identification of Neglected Lungworms and Heartworms Affecting Cats. J. Clin. Microbiol..

[57] Euzeby J. (1981). Helminthes para-sites de l’appareil respiratoire. Diagnostic Expérimental des Helminthoses Animales.

